# Effect of Solution Treatment on Precipitation Behaviors, Age Hardening Response and Creep Properties of Elektron21 Alloy Reinforced by AlN Nanoparticles

**DOI:** 10.3390/ma10121380

**Published:** 2017-12-02

**Authors:** Abdollah Saboori, Elisa Padovano, Matteo Pavese, Hajo Dieringa, Claudio Badini

**Affiliations:** 1 Department of Applied Science and Technology (DISAT), Politecnico di Torino, Corso Duca Degli Abruzzi 24, 10129 Torino, Italy; Elisa.padovano@polito.it (E.P.); Matteo.pavese@polito.it (M.P.); Claudio.badini@polito.it (C.B.); 2 Helmholtz-Zentrum Geesthacht, Magnesium Innovation Centre—MagIC, Max-Planck Street 1, 21502 Geesthacht, Germany; hajo.dieringa@hzg.de

**Keywords:** Elektron21, solution treatment, metal matrix nanocomposites, creep, hardness

## Abstract

In the present study, the solution and ageing treatments behavior of Mg-RE-Zr-Zn alloy (Elektron21) and its nano-AlN reinforced nanocomposites have been evaluated. The properties of the thermal-treated materials were investigated in terms of Vickers hardness, the area fraction of precipitates, microstructure and phase composition. The solution treatments were performed by treating at 520 °C, 550 °C and 580 °C in argon atmosphere. The outcomes show that the hardness of the solutionized alloys was slightly affected by the solution temperature. X-ray diffraction and image analysis revealed that the complete dissolution of precipitates was not possible, neither for Elektron21 (El21) nor for its AlN containing nanocomposites. The ageing treatment of El21 led to a significant improvement in hardness after 20 h, while for longer times, it progressively decreased. The effect of ageing on the hardness of El21–AlN composites was found to be much less than this effect on the hardness of the host alloy. Electron backscatter diffraction (EBSD) analysis of El21 and El21–1%AlN after solution treatment confirm the random orientation of grains with a typical texture of random distribution. The as-cast creep results showed that the incorporation of nanoparticles could effectively improve the creep properties, while the results after solution treatment at 520 °C for 12 h followed by ageing treatment at 200 °C for 20 h confirmed that the minimum creep rate of T6-El21 was almost equal to the as-cast El21–AlN.

## 1. Introduction

Metal matrix composites (MMCs) reinforced by nanoparticles have been the subject of several studies over the last years due to their promising properties. Indeed, the incorporation of nano-sized particles as reinforcement in a metal matrix could considerably improve the mechanical properties of conventional alloys such as strength, stiffness, creep resistance and fatigue strength as well as their specific physical and/or chemical properties. It could enhance the performances of many traditional metallic materials, thus extend their functional and structural applications [1,2,3,4,5].

Among the metal matrix nanocomposites, magnesium matrix composites have raised an increasing interest in the scientific community and nowadays have found many applications in modern industries. In fact, the low density of magnesium (two-thirds of aluminum and one-fifth of steel) makes Mg alloys very attractive in automotive, aerospace and electronics industries [6,7,8,9,10]. However, the relatively low strength and modulus, as well as scarce creep behavior, of conventional Mg alloys, in particular at high temperatures, limit their applications. Nonetheless, recently, there has been a growing interest in magnesium alloys for high-temperature applications such as gearbox casings for the helicopter industry. Moreover, a European research program “Magnesium for Aerospace Applications” (FP6 AEROMAG) has promoted the collaboration between a number of magnesium component producers and the aerospace industry with the aim of new magnesium alloys development and introducing new manufacturing processes for aerospace applications [11]. Thus, Mg–rare earth (RE) alloys with superior mechanical properties (over conventional Mg alloys) at room and high temperatures have been developed [12,13]. Indeed, the rare earth elements play a key role in the improvement of creep properties, the thermal stability of microstructure and mechanical properties of magnesium alloys. Among the Mg-RE alloys, Mg-RE-Zr-Zn alloy (Elektron21), which is a commercial alloy containing Nd, Gd, Zn, and Zr, is one of the most widely used magnesium alloys for high-temperature permanent-mold and sand-casting applications up to 200 °C. The specific composition of this alloy was optimized to overcome the main limits of the currently available commercial magnesium alloys (WE43). The presence of neodymium, which presents a low solid solubility limit in magnesium, allows for an improvement of the strength, especially at high temperature. Gadolinium, similar to neodymium, shows a reduced solid solubility when the temperature progressively decreases, thus promoting the precipitation strengthening effect [8,14]. The addition of neodymium to a magnesium alloy containing gadolinium reduces the solid solubility of the latter favoring the precipitation hardening mechanism [8,14,15]. Lyon et al. [8] reported that the use of both these elements improves the castability in terms of microshrinkage. The presence of zinc in a small amount (up to 1.3%) leads to an enhancement of the mechanical strength without reducing the ductility. In addition, a lower zinc level in Mg-Nd alloy, such as in the case of El21, can modify the response of the alloy to ageing [8,16]. The presence of zirconium allows obtaining a fine grains structure resulting in an improvement of mechanical properties, castability, and corrosion resistance [8,14]. Thus, the strength of Mg-RE alloys is obtained essentially through the precipitation strengthening.

On the other hand, it is reported that a significant mechanical improvement can be achieved through the incorporation of only small amount of nanoparticles [17]. In fact, the addition of a low amount of ceramics nanoparticles has the aim to create local reinforcements in the base alloy. In general, the strengthening effect through the addition of nanoparticles can be achieved through different mechanisms, including load transfer, Hall–Petch strengthening, thermal expansion coefficient mismatch and Orowan looping [18]. Load transfer effect is the first possible mechanism of strengthening in metal matrix nanocomposites. In the mechanism, the load transfers from the soft matrix to high strength reinforcement. It has been observed that this mechanism depends seriously on the interface of metal/reinforcement particles. Moreover, according to the Hall–Petch strengthening equation, the grain refinement that can be achieved through the addition of nanoparticles can also participate in the strengthening of the alloy. It also is reported that the dispersion of nanoparticles in the metallic matrix results in the so-called Orowan strengthening effect. The nanoparticles indeed act as non-shearable obstacles that hinder the motion of the dislocations.

The effect of different nanoparticles on the processing and on the mechanical properties of these alloys have been previously investigated [19]. Among them, the addition of nano aluminum nitride (AlN) was the object of only a few investigations and further studies are needed to be undertaken to explore the potential of this nanoparticles as reinforcement in the El21 alloy [17].

The main aim of this work is to study the influence of AlN nanoparticles incorporation on the microstructure, solution–precipitation behavior and creep properties of El21 magnesium alloy. For this purpose, the El21 alloy, with and without AlN nanoparticles, was carefully produced via an ultrasound-assisted casting technique. Thereafter, the solution–precipitation behavior of the nanocomposite and base alloy was evaluated by means of hardness measurements. In addition, minimum creep rate of El21 and its nanocomposites was compared before and after solution treatment to assess the effect of nanoparticles incorporation.

## 2. Materials and Methods

### 2.1. Composite Fabrication

El21 magnesium alloy and El21 composite reinforced by aluminum nitride (AlN) nanoparticles were produced by an ultrasound assisted casting technique. For this reason, a commercial El21 alloy was melted in a mold, kept at 720 °C for 1 h, and then AlN nanoparticles containing 20% pure aluminum as an impurity were incorporated into the molten El21 at 670 °C under the flux of argon + 1% SF_6_. Thereafter, the AlN nanoparticles (20–30 nm) were dispersed within the molten El21 by means of mechanical stirring (200 rpm) followed by ultrasonication for 5 min at 0.3 kW. In fact, these parameters of ultrasonication and stirring are the optimum parameters of dispersion. After ultrasonication, the mold with molten alloy was lowered into a water bath very quickly. The chemical composition of the commercial El21 is given in Table 1.

### 2.2. Heat Treatment

**Figure 1:**
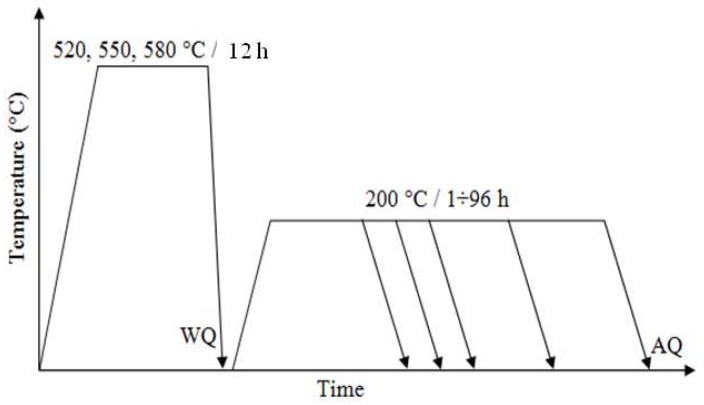
Schematic diagram indicating the heat treatment cycles used in the present investigation.

The as-cast samples were solutionized at three different temperatures (520 °C, 550 °C and 580 °C) for 12 h and then quenched into hot water (80 °C). Afterwards, the samples were aged at 200 °C for 1–96 h in an oil bath and then cooled in the air (Figure 1).

### 2.3. Characterization

#### 2.3.1. Microstructural Characterization

To study the microstructure of specimens by an optical microscope (OM), scanning electron microscope (SEM) and electron backscatter diffraction (EBSD), they were mounted in resin and ground with SiC papers down to 4000 grit size and thereafter by diamond paste (down to 1 µm). Finally, a 9% picric acid solution was used as an etchant. A field emission scanning electron microscope (FESEM; Merlin-Zeiss, Munich, Germany, operating at 15 kV) equipped with an energy-dispersive X-ray spectrometer (EDS) was employed for microstructural evaluations. The microstructure of El21 and El21–AlN solution treatment was analyzed by EBSD in a focused ion beam field emission gun scanning electron microscope (FIB-FEGSEM, Helios NanoLab 600i FEI, OR, USA,) equipped with an Oxford-HKL EBSD system operated at a voltage of 15 kV and using a beam current of 2.7 nA. X-ray diffraction (XRD) patterns of as-polished samples were recorded on a Philips X’Pert Diffractometer using Cu-Kα radiation. Sample preparation for XRD analysis included grinding the samples with the height of less than 5 mm down to 4000 grit size SiC paper, washing, and drying. The image analysis process to calculate the area fraction of precipitates has been carried out by an image analysis software (V. 1.48, NIH, MD, USA) on 30 images for each specimen. This image analysis process, according to a previous work, includes acquiring images, normalizing, shadow correction and binarization [14].

#### 2.3.2. Mechanical Properties

Hardness measurements were carried out on as polished surfaces with a load of 5 kg and the dwell time was 15 seconds. The hardness results are the average of 5 measurements for each sample. Compression creep tests were carried out at 240 °C and constant stresses between 80 MPa and 120 MPa on cylinders with a length of 15 mm and a diameter of 6 mm. Deformation as a function of time was recorded and the first derivative was calculated to yield the minimum creep rate (έ*_s_*).

## 3. Results and Discussion

### 3.1. Elektron21

#### 3.1.1. As Cast

**Figure 2:**
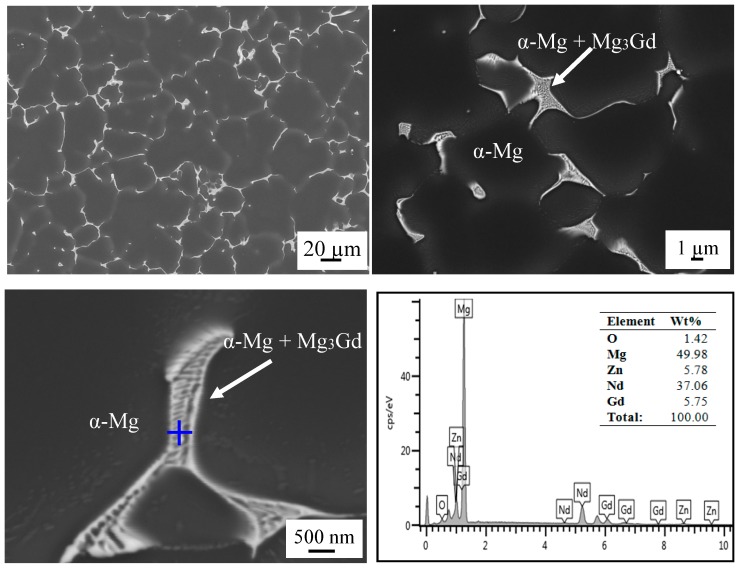
Microstructure and corresponding EDS analysis of El21 as-cast alloy.

To evaluate the properties of as-cast El21, a preliminary characterization has been performed. Figure 2 shows the as-cast microstructure of El21 which contains two different areas: α-Mg solid solution and α-Mg + Mg_3_(Nd,Gd) eutectic phase on the grain boundaries. Mg_3_(Nd,Gd) phase is a modification of Mg_3_Nd phase where neodymium is partially replaced by gadolinium without modifying the crystal structure. This substitution does not damage the crystal structure because of a small difference in the atomic radii between gadolinium r_Gd_ = 0.1802 nm and neodymium r_Nd_ = 0.1821 nm [20].

**Figure 3:**
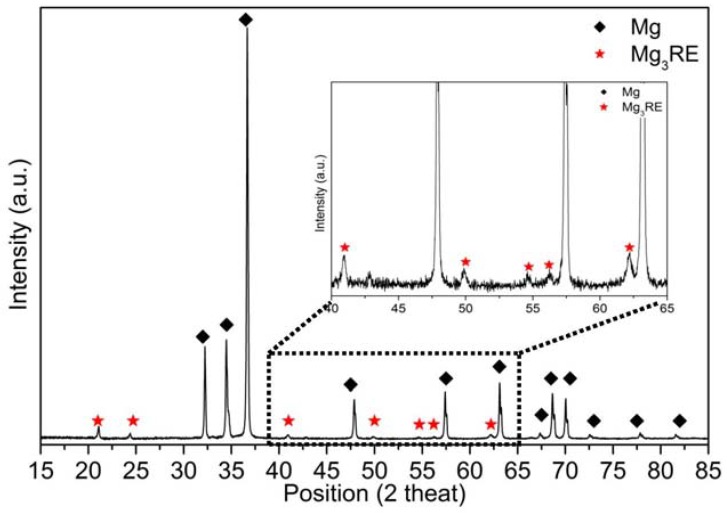
XRD spectra of as-cast El21.

**Figure 4:**
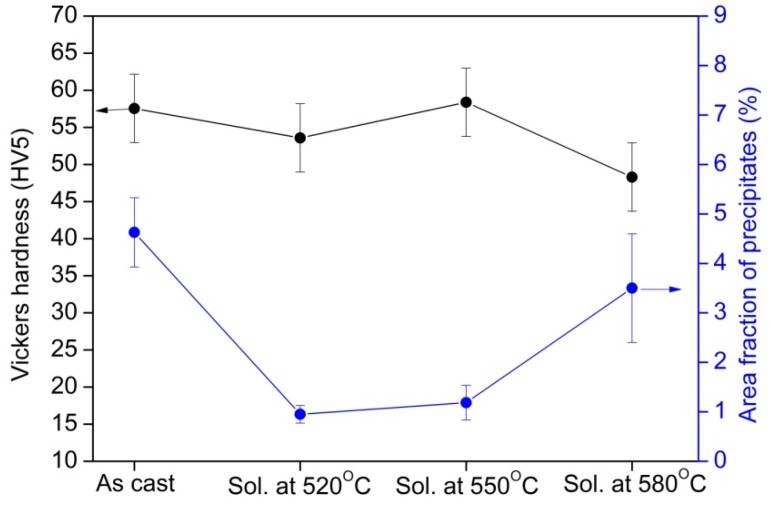
Vickers Hardness and area fractions of precipitates for as-cast El21 and solutionized samples at different solution treatment temperatures.

The XRD measurements on the as-cast EL21 identified the main peaks of Mg (the matrix phase). In addition, the peaks corresponding to Mg_3_RE phase as the main intermetallic phase (Figure 3) were identified. As expected, in as-cast condition, the El21 alloy already displays a high density of intermetallic precipitates. The area fraction analysis of precipitates, realized by means of image analysis, is presented in Figure 4 and demonstrates the presence of almost 5 vol.% precipitates in the as-cast El21.

#### 3.1.2. Solution Treatment

**Figure 5:**
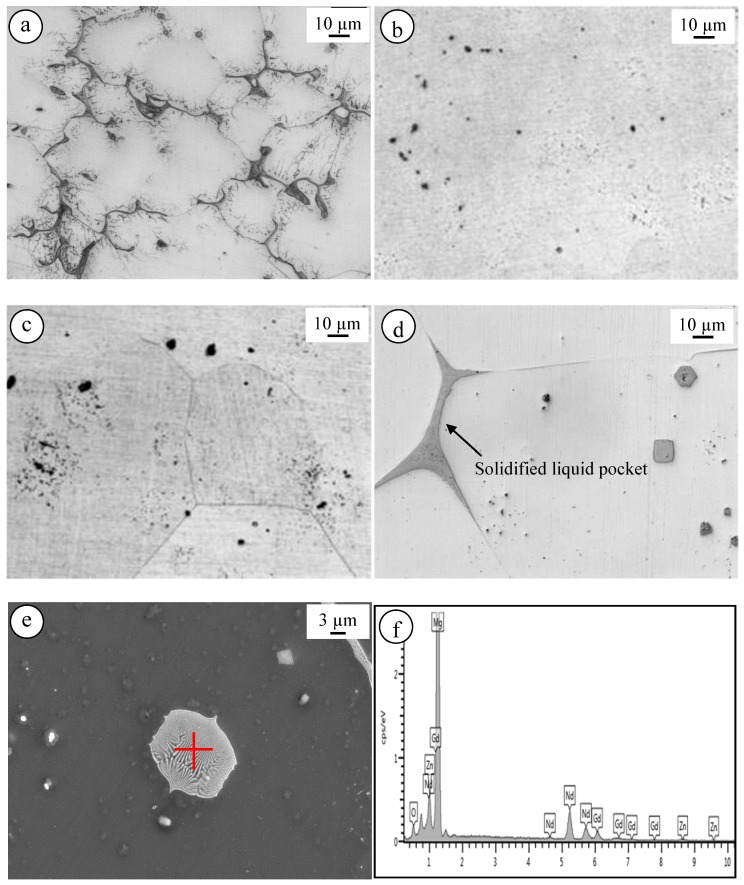
The microstructure of: (**a**) El21 as-cast; and solutionized at: (**b**) 520 °C; (**c**) 550 °C; and (**d**–**f**) 580 °C and EDS corresponding to the hexagonal eutectic cell shown in (**e**).

As mentioned earlier, to find the best experimental conditions for dissolution of precipitates in El21 alloy, different solution treatments were performed by solutionizing at 520 °C, 550 °C and 580 °C in an argon atmosphere followed by quenching in hot water. The properties of as-solutionized specimens in terms of Vickers hardness, microstructure, and phase composition have been compared to as-cast samples. The hardness, as shown in Figure 4 for the El21 alloy, is reduced in as-solutionized samples, a consequence of the dissolution of precipitates at 520 °C and 550 °C, and, in the case of 580 °C, melt formation at grain triple points and within the grains (overheating effect). It can be seen in Figure 5, by increasing the solutionizing temperature there is an increase not only in the grain size but also in the size of residual precipitates, both at the grain boundaries and inside the grains. Figure 4 also shows that the area fraction of the intermetallic phase (Mg_3_(Nd,Gd)) significantly decreases after solution treatment because of dissolution of intermetallic in the magnesium matrix. In as-cast condition, the area fraction of the precipitates is about 5% and this value decreased considerably down to 1.0% and 1.3% and 3.0% after solution treatment at 520 °C, 550 °C and 580 °C, respectively. The behavior at 580 °C, where the area fraction of precipitates increases with respect to samples solutionized at a lower temperature, is attributed to the source of calculations. Indeed, in the evaluation of area fraction of precipitates at 580 °C, the solidified pockets after overheating at this temperature was also considered in the calculations. However, it should be noticed that the aim of area fraction analysis in this work was a rough qualitative analysis, not a quantitative one. These solidified pockets at 580 °C, together with the significant grain growth observed, could affect the hardness of samples after solution treatment so that the hardness of EL21 solutionized at 580 °C is lower than the hardness of the other solutionized samples (Figure 4). It must be noted that the hardness at 550 °C is higher than the hardness at 520 °C and even higher than the hardness of as-cast alloy; this could be attributed to the presence of small new precipitates at 550 °C that hinder the movement of dislocations and leads to the hardness improvement. However, overheating because of solutionizing at 580 °C results in the killing of hardening response of the alloy. In fact, according to the solubility limits of the alloying elements in EL21 alloy, and to the real composition of the alloy under investigation, it can be concluded that it is not possible to achieve the complete dissolution of Nd-based precipitates since the Nd content in the present alloy is more than the solubility limit of Nd. On the contrary, the complete dissolution of Gd-based precipitates seems possible since the solubility limit of this element is well over its content in the present alloy.

To study the phase composition after solution treatment at various temperatures, EDS analysis was employed. Figure 5a–d shows the microstructure of El21 in as-cast condition and after solution treatment at 520 °C, 550 °C, and 580 °C, respectively. Figure 5e,f presents SEM images of a solidified liquid pocket present in the sample after solution treatment at 580 °C and its corresponding EDS. The composition of this eutectic cell is 90 at.% Mg, 2.5 at.% Zn and 7.5 at.%. rare earth (5.5 at.% Nd, 2.0 at.% Gd). Due to the intrinsic EDS resolution limits, no further discrimination of these precipitates was possible. Figure 5d shows that solutionizing at very high-temperature results in the formation of the liquid phase at the grain triple points and thereafter this liquid solidifies in eutectic cells. In fact, it can be concluded that solutionizing at temperatures higher than the eutectic temperature leads to decreasing the precipitation hardening response of the alloy.

**Figure 6:**
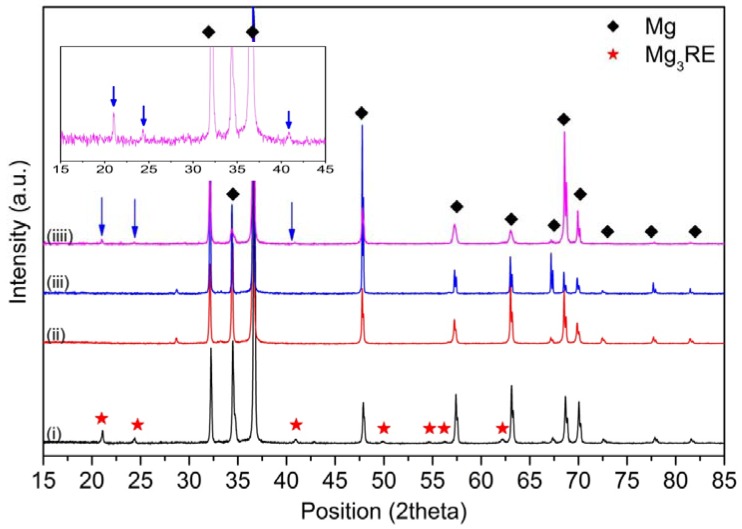
XRD of: (i) El21-as cast; and El21 solutionized at: (ii) 520 °C; (iii) 550 °C; and (iiii) 580 °C.

XRD measurements were carried out on solutionized El21 at different temperatures to compare the phase composition after the solution treatment at different temperatures (Figure 6).

Figure 6 shows that the Mg_3_(Nd,Gd) intermetallic phase was detected in the as-cast pattern, while this experiment did not detect any evidence of this phase in the samples after solution treatment at 520 °C and 550 °C. These results provide further support for the hypothesis that most of the intermetallic phase dissolved during the solution treatment. In fact, as shown before, not all the precipitates dissolved during the solution treatment, but the amount of residual precipitate was lower than the resolution of the XRD measurement.

On the contrary, in the solutionized sample at 580 °C, the peaks corresponding to Mg_3_RE are clearly detectable (blue arrows), confirming the formation of new eutectic cells at this temperature as a consequence of overheating. Indeed, at this temperature, the solidified regions can be detected at the grain triple points as well as a new Mg_3_RE phase which is formed at this temperature and does not contribute to the hardness increment.

#### 3.1.3. Ageing Treatments

**Figure 7:**
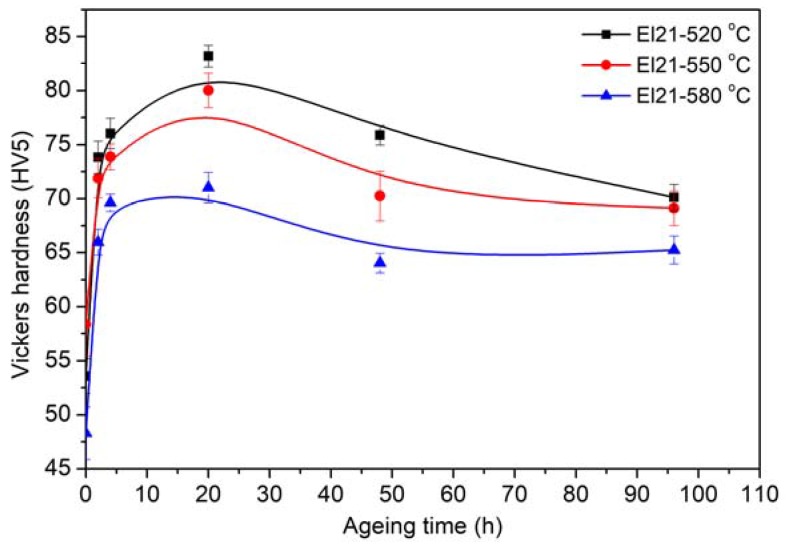
Hardness variation of El 21 solutionized at 520 °C, 550 °C and 580 °C then aged at 200 °C.

Figure 7 displays the hardening curves of El21 after solution treatment at different temperatures and isothermal ageing treatment at 200 °C for different periods of time. As explained earlier, after solution treatment, Mg3(Gd,Nd) intermetallic phase partially dissolves in the matrix and thereafter, during the ageing step, these intermetallic precipitates again with a very fine grain. The curves show how at first the hardness increased significantly, reaching its maximum value after 20 h, thereafter further ageing led to a decrease of the hardness value due to the over-ageing effect. Previous research has established the precipitation sequence of the Mg-Gd series alloy as follow: S.S.S.S–β″(Mg3Gd)–β′(Mg7Gd)–β1(Mg3Gd)–β(Mg5Gd) [21,22].

According to the literature, coexistence of coherent β″ and semi-coherent β′ intermetallic phases could be responsible for maximum hardening effect after 20 h of isothermal ageing, while β phases formation leads to decreasing the hardness at longer ageing times [22,23]. This behavior was observed in all the samples obtained at the three solutionizing temperatures, but the maximum peak hardness was obtained after solutionizing at 520 °C and 20 h ageing at 200 °C. In fact, the decrease of maximum hardness by increasing the solutionizing temperature can be explained both by the magnesium grain growth and by the fact that the amount of residual precipitates left after solutionizing grows with the increasing temperature. In fact, the samples obtained at the higher solutionizing temperatures contain less alloying elements in solid solution, so that their ageing is less effective.

**Figure 8:**
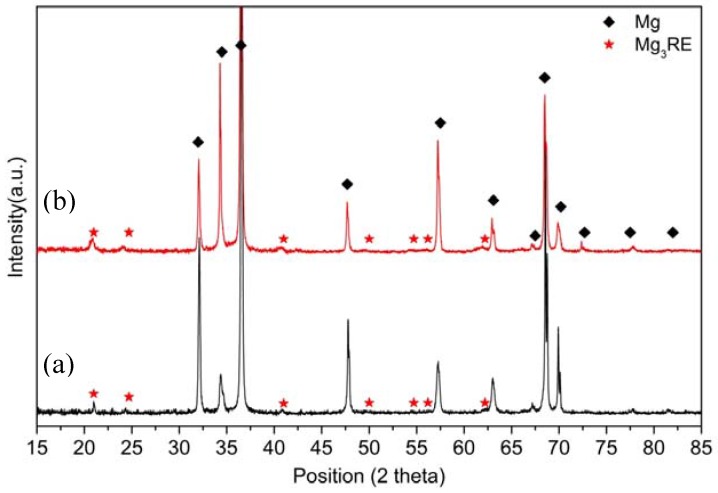
XRD spectra of EL21: (**a**) after solutionizing at 580 °C; and (**b**) solutionizing at 580 °C followed by ageing at 200 °C for 96 h.

Figure 8 compares the X-ray diffraction patterns of EL21 after solution treatment at 580 °C and solution treatment at 580 °C followed by ageing for 96 h. It is evident that the peaks corresponding to the Mg_3_RE intermetallic phase are present in both patterns. This confirms that the ageing treatments, even after longer periods, did not modify the phase composition of the alloy.

### 3.2. Elektron21–AlN Composites

#### 3.2.1. As cast El21–AlN Composites

**Figure 9:**
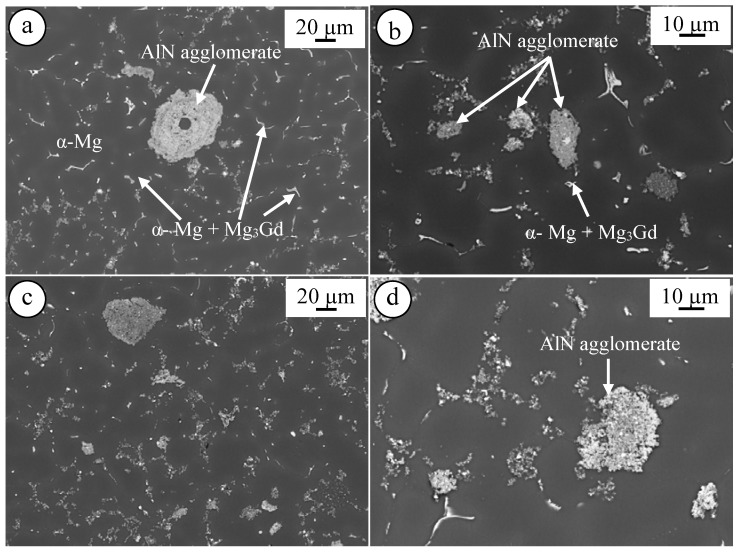
SEM images of as-cast: (**a**,**b**) El21–1.0 wt.% AlN; and (**c**,**d**) El21–2.0 wt.% AlN.

Figure 9 shows the microstructure of as-cast El21–1.0 wt.% AlN (Figure 9a,b) and as cast El21–2.0 wt.% AlN (Figure 9c,d). As can be seen in both cases, similar to the base alloy, the microstructure is the combination of magnesium matrix dendrites separated by interdendritic regions. Moreover, even if the dispersion of AlN within the Mg matrix seems to be rather uniform, some big AlN clusters are visible in both nanocomposites with different AlN content.

**Figure 10:**
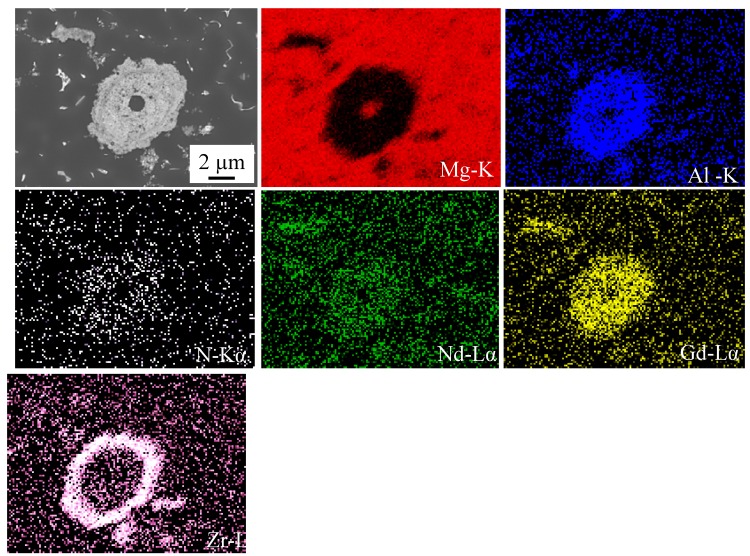
EDS mapping of El21–1.0 wt.%AlN nanocomposite near to a big agglomerate.

Shi et al. [23] reported a poor adhesion between magnesium and AlN and this poor adhesion resulted in particle pushing and finally formation of big AlN clusters. As can be seen in Figure 9b,d, it seems most of the nanoparticles were pushed into the eutectic regions during the solidification. SEM mapping was carried out in the El21–1.0 wt.% AlN nanocomposites near to a very dense agglomerate of AlN, and the results are shown in Figure 10. It seems Zr reacted with AlN on the surface of the cluster, and this reaction was reported in the literature. He et al. reported that AlN may be involved in a reaction with Zr to form ZrAl_3_ or ZrAl_2_ at their interface [24]. On the other hand, William et al. reported instead that Zr atoms can be absorbed on the surface of aluminum nitride and as a consequence ZrN forms at the interface of the metal matrix and AlN particles. This reaction interfacial bonding was shown to result in the improvement of the final properties of nanocomposite [25].

#### 3.2.2. Solution Treatment

**Figure 11:**
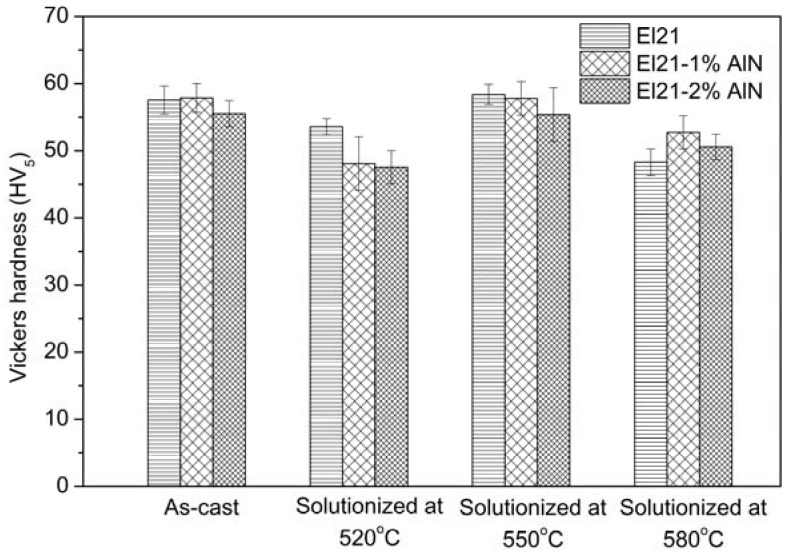
Hardness values of El21, El21–1.0 wt.% AlN and El21–2.0 wt.% AlN as cast and solution treated at different temperatures.

Figure 11 shows the hardness results of El21–1.0 wt.% AlN and El21–2.0 wt.% AlN in as-cast condition and solutionized at 520 °C, 550 °C and 580 °C.

**Figure 12:**
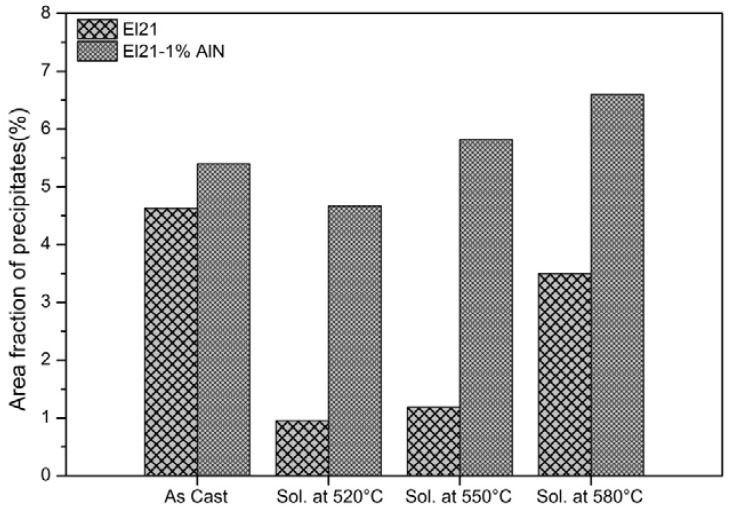
The area fraction of precipitates in El21 and El21–1.0 wt.%AlN after casting and solution treatment at different temperatures.

As can be seen, the hardness of nanocomposites was reduced with the treatment at 520 °C, did not change significantly after solution treatment at 550 °C and was again slightly reduced after treatment at 580 °C. The behavior is not significantly different from the one of the base alloy, as shown in the previous section. If the hardness trend is similar between the El21 alloy and the composites, the area fraction of precipitates after solution treatment at different temperatures shown in Figure 12 is rather different for the cases of El21 and El21–1.0 wt.% AlN. This figure shows evidently that the number of precipitates after solutionizing, measured by image analysis, is still very high in the composites. A possible explanation for these results could be found in the composition of the precipitates that is different in the case of the base alloy and of the composites. In particular, the formation of Al–RE precipitates is observed in the composites during the casting, and these precipitates seem very stable and are not dissolved even at 580 °C.

**Figure 13:**
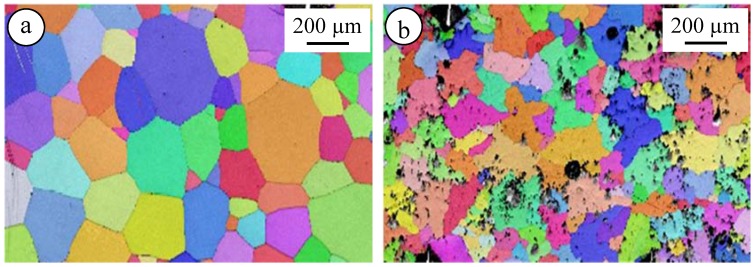
EBSD images of: (**a**) El 21; and (**b**) El21–1%AlN after solution treatment at 520 °C.

The fact that the microstructure of material is only limitedly altered by the addition of AlN nanoparticles is also demonstrated by the EBSD images of El21 and El21–1 wt.% AlN after solution treatment at 520 °C, as shown in Figure 13. Randomly oriented grains with an average grain size of 110 ± 9 µm and 95 ± 7 µm for El21 and El21–1%AlN, respectively, measured using grain size distribution from EBSD results, are observed in these images. The texture of both samples was similar to a random distribution, which is the typical texture of materials obtained by casting.

**Figure 14:**
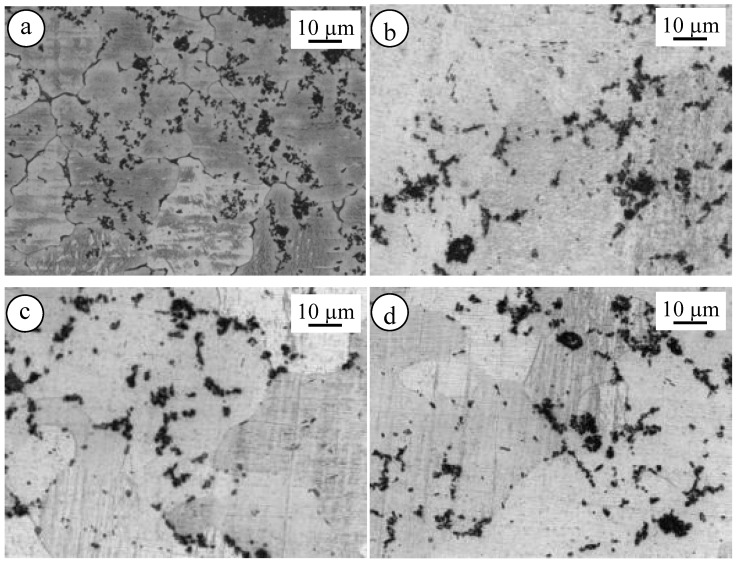
Optical microscopy images of El 21–1.0 wt.% AlN: (**a**) as-cast; and solutionized at: (**b**) 520 °C; (**c**) 550 °C; and (**d**) 580 °C.

**Figure 15:**
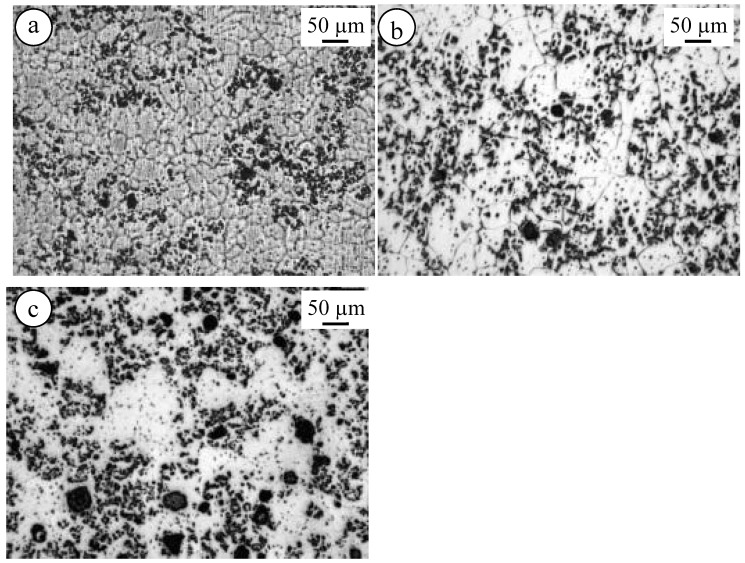
Microstructure of El21–2.0 wt.% AlN: (**a**) as-cast; (**b**) solutionized at 520 °C; and (**c**) solutionized at 550 °C.

Figure 14 shows the microstructure of El21–AlN (Figure 14a) as-cast and solutionized at 520 °C (Figure 14b), 550 °C (Figure 14c) and 580 °C (Figure 14d). In this case, the microstructure of the El21–AlN nanocomposites is changed during the solution treatment. Apparently, grain size increases, in particular, when increasing the solution treatment. The same effect was observed in the case of El21–2.0%AlN after solution treatment at 520 °C, 550 °C, and 580 °C, as presented in Figure 15.

**Figure 16:**
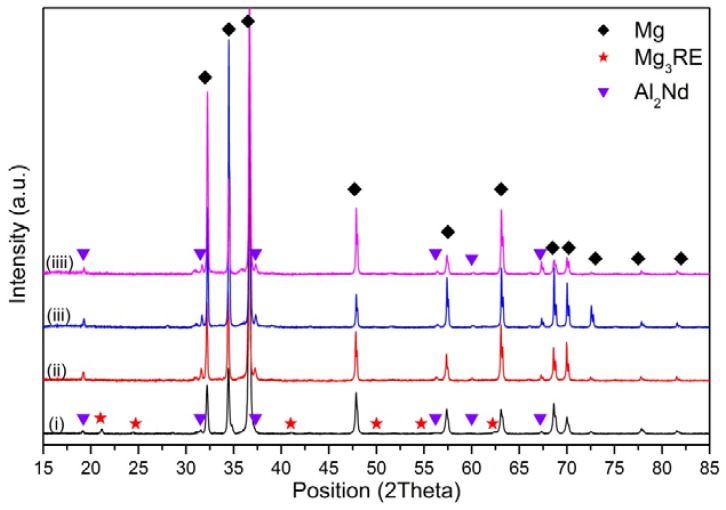
X-ray diffraction patterns of EL21–1.0 wt.% AlN: (i) as cast; and solutionized at: (ii) 520 °C; (iii) 550 °C; and (iiii) 580 °C.

Figure 16 shows the X-ray diffraction patterns of El21–1.0 wt.% AlN nanocomposites. In this case, after solution treatment and ageing, the peaks of Al_2_Nd phase are observed. This means that part of Al introduced as an impurity with AlN particles reacts with Nd and forms Al_2_Nd, which is very stable at high temperature and does not dissolve during the solution treatment. This compound is present in all El21–1.0 wt.%AlN samples, as cast and solution treatment at different temperatures; however, while in as-cast samples Mg_3_RE is also found, in the solutionized and aged samples, only Al_2_Nd peaks are observed. This suggests that Mg_3_RE partially dissolves during the solution treatments starting from 520 °C, while Al_2_Nd does not dissolve.

**Figure 17:**
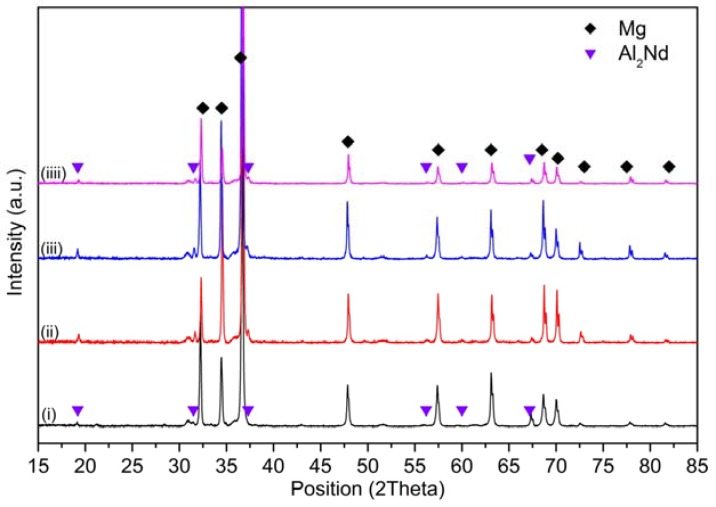
X-ray diffraction patterns of EL21–2 wt.% AlN: (i) as-cast; and solutionized at: (ii) 520 °C; (iii) 550 °C; and (iiii) 580 °C.

In addition, the X-ray diffraction patterns of El21–2.0 wt.% AlN as-cast and solutionized at 520 °C, 550 °C and 580 °C are similar to those in the case of El21–1.0 wt.% AlN: in addition to the Mg phase, peaks of Al_2_Nd are present. Moreover, it is also evident that the Mg_3_RE peaks which were identified in as-cast El21–2.0 wt.% AlN pattern were not present in the patterns of El21–2.0 wt.% AlN after solution treatment (Figure 17).

#### 3.2.3. Ageing Treatment

**Figure 18:**
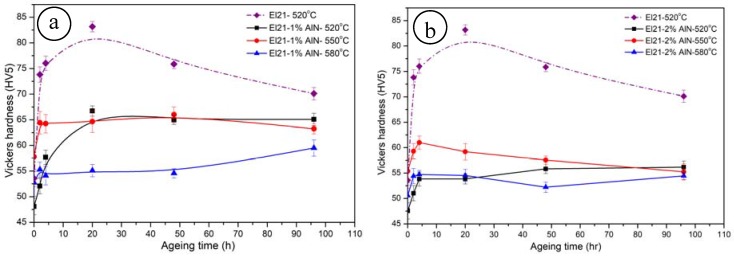
Hardness variation of: (**a**) El21–1.0 wt.% AlN; and (**b**) El21–2.0 wt.% AlN solutionized at 520 °C, 550 °C and 580 °C then aged at 200 °C for various periods.

In the case of El21–AlN nanocomposites, the ageing has a much smaller effect on the hardness with respect to the pure El21. Nonetheless, it is reported that the addition of ceramic particulates to metallic alloys does not qualitatively change their precipitation sequence but changes precipitation and dissolution kinetics up to an extent, depends on the specific phase. However, it has also been observed that the composite ages markedly faster than unreinforced alloy [26]. It is possible to see in Figure 18 for El21–1.0 wt.% AlN and El21–2.0 wt.% AlN how the maximum hardness is slightly improved with respect to the solutionized samples and does not increase over the as-cast condition.

As mentioned before, in the case of nanocomposites, there are some problems in the solutionizing of the precipitates. Since rare earth based precipitates are partially dissolved, the improvement of hardness is very low after ageing. Moreover, in the nanocomposites, the presence of AlN agglomerates could further reduce the effect of hardening of the base alloy.

It can be also seen that the hardening response from the thermal treatment is neither a linear function of the solution temperature nor the same as the base alloy. The hardness increases significantly in the first hours, and thereafter it remains stable. Moreover, it can be observed that the nanocomposites do not present a significant strengthening response during the ageing process, probably due to the limited dissolution of the precipitates during the solution step and presence of nano particles. However, as discussed earlier, it is not possible to improve the dissolution of precipitates, so that no significant increase of hardness after ageing can be obtained with the composites. It is believed that the significant difference between solid state reactions in the nanocomposite and monolithic alloy arise from the largely different thermal coefficient of thermal expansion of reinforcement and the host alloy. Indeed, thermal mismatch between matrix and nanoparticles results in the increase of dislocation concentration near the matrix/particle interface which, as a consequence, causes enhanced nucleation rate and therefore both heterogeneous precipitation and evolution of precipitates [26].

**Figure 19:**
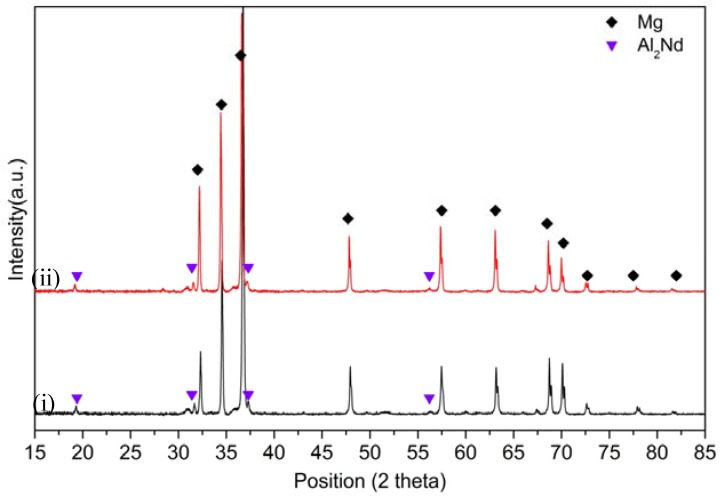
X-ray diffraction patterns of El21–2.0 wt.% AlN: (i) as-solution treated at 580 °C; and (ii) solution treated followed by ageing at 200 °C for 96 h.

Figure 19 displays the X-ray diffraction patterns of EL21–2.0 wt.% AlN: (i) as-solution treated at 580 °C; and (ii) solution treated followed by ageing at 200 °C for 96 h. As shown in this image, there is no evidence regarding changes in phase composition, and Al_2_Nd, which forms during the casting, is still present in the composite even after solution treatment at high temperatures and long periods of ageing.

### 3.3. Creep Properties

To study the effect of the best T6-heat treatment (solution treatment at 520 °C for 12 h followed by ageing treatment at 200 °C for 20 h) on the creep properties of El21 and El21–AlN nanocomposite, compression creep tests were performed at 240 °C and stresses within 80–120 MPa.

**Figure 20:**
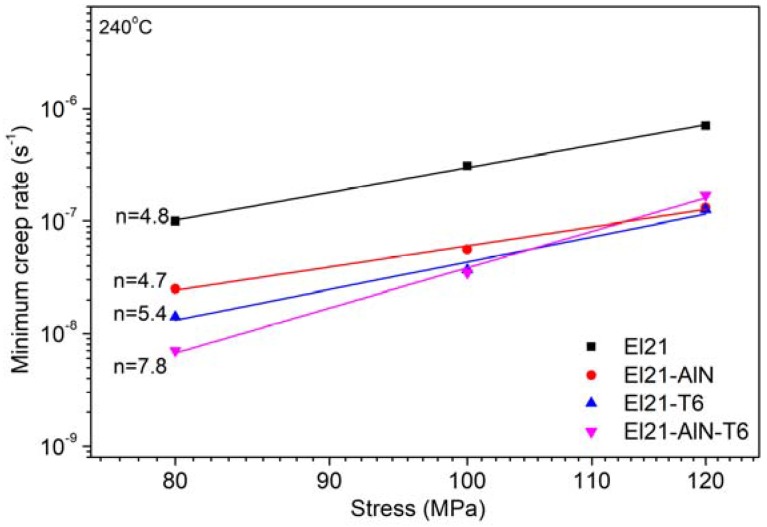
Double logarithmic plot of minimum creep rate as a function of applied stress.

The Norton–Arrhenius equation explains the relationship between the minimum creep rate (έ), temperature (T) and applied stress (σ) [17].

(1) $${έ}_{s}=\frac{ADGb}{kT}{\left(\frac{\sigma }{G}\right)}^{n}$$

where *A* is a material-dependent constant, *G* is the shear modulus, *b* is the burgers vector, *k* is the Boltzmann constant, *n* is the stress exponent and *D* is the diffusion coefficient. In fact, the stress exponent gives information regarding the rate-controlling deformation mechanisms during creep. The stress exponent *n* can be identified through the plotting the minimum creep rates versus applied stress (in double logarithmic). Figure 20 shows the double logarithmic plot of minimum creep rate as a function of applied stress. As can be seen in this plot, the stress exponent *n* is 4.8 and 5.4 for as-cast and as-T6 El21 alloy, respectively. Moreover, it is clear that the El21–AlN nanocomposite in both cases (as-cast and as T6) shows lower minimum creep rate with respect to the as-cast El21. These results are likely to be related to the effect of nanoparticles and precipitates on hindering the dislocation motion that could result in the lower minimum creep rate. It is reported that, in the strengthening of metallic matrix by particle, lattice dislocations are forced by particles to bow or pile up, and their movement needs an external stress dependent on a microstructural parameter. For instance, the mean free inter-particle distance strongly affects the mechanical strength of composites so that smaller inter-particle distances result in higher mechanical strength [27].

On the other hand, the lower minimum creep rate of El21 after T6-treatment compared to the as-cast one could be attributed only to the interaction of dislocation with the homogeneously dispersed precipitates after the ageing treatment. Among the several possible explanations for these results, the most probable ones are: (i) Orowan looping, which causes an additional stress to bow the dislocation around the nanoparticles or precipitates [28]; (ii) additional stress for detaching a dislocation from the particles or precipitates [29]; and (iii) additional stress for dislocation climb over an obstacle such as nanoparticles or a precipitate [30]. According to the literature, a value of *n* = 5.0 is attributed to the dislocation climbing at high temperatures as the rate-controlling deformation mechanism [17]. Moreover, in the case of El21–AlN nanocomposite after solution-aging treatment, the value of *n* = 7.8 can be related to a change of creep mechanism to cross-slip with respect to the other specimens.

In the case of El21–AlN after T6 treatment, it could be noticed that, due to the presence of nanoparticles, the type of precipitation was changed from homogeneous to heterogeneous precipitation. This may affect the minimum creep rate: the stress for dislocation motion increases and, as a consequence, a lower minimum creep rate can be achieved.

## 4. Conclusions

Solution and ageing treatment behavior of El21 alloy and El21–AlN composites have been investigated in the present study. The main conclusions can be drawn as follow:

(1) As-cast El21 showed the typical behavior of age hardening in the magnesium alloys. The best condition for solution treatment and ageing process was solutionizing at 520 °C for 12 h followed by ageing at 200 °C for 20 h.
(2) Solution treatment at very high temperatures (higher than eutectic) brings the microstructure to a point where there are liquid regions at grain triple points as well as within the grains, and consequently kills the precipitation hardening response.
(3) Through the comparison between as-cast and as-solutionized El21, together with the variation of the area fraction of precipitates, it could be concluded that the complete dissolution of precipitates during the solution treatment was not possible and the residual precipitates grew at higher solution treatment temperatures.
(4) In the case of the El21–AlN nanocomposite, precipitation of Al_2_Nd was detected in the solutionized case, while, in the case of El21, the only Mg_3_RE phase was identified as the main intermetallic phase.
(5) The El21–AlN composites showed a similar hardness with respect to El21 alloy after solution treatment, but gained a very limited hardness during the early stage of ageing treatment and remained unchanged up to an ageing treatment of 96 h.
(6) By increasing the solutionizing temperature, the maximum hardness decreases, which could be related to the magnesium grain growth and the growth of residual precipitates left after solutionizing with the increasing temperature. In fact, the samples obtained at the higher solutionizing temperatures contain less alloying elements in solid solution, so that their ageing is less effective.
(7) The first creep results confirmed that the addition of nanoparticles could be effective to improve the creep properties, while the results after the T6 treatment showed that the minimum creep rate of T6 El21 was almost equal to the as-cast El21–AlN.

## Figures and Tables

**Table 1:** Chemical composition of (wt.%) of El 21 Mg-alloy billets.

-	Mg	Nd	Gd	Zr	Zn
wt.%	Balance	2.6–3.1	1.0–1.7	0.49	0.2–0.5
